# Impact of Over-the-Counter Restrictions on Antibiotic Consumption in Brazil and Mexico

**DOI:** 10.1371/journal.pone.0075550

**Published:** 2013-10-16

**Authors:** Yared Santa-Ana-Tellez, Aukje K. Mantel-Teeuwisse, Anahi Dreser, Hubert G. M. Leufkens, Veronika J. Wirtz

**Affiliations:** 1 WHO Collaborating Centre for Pharmaceutical Policy & Regulation, Utrecht Institute for Pharmaceutical Sciences (UIPS), Utrecht, The Netherlands; 2 Center for Health Systems Research, National Institute of Public Health, Cuernavaca, Mexico; 3 Center for Global Health and Development, Boston University, Boston, Massachusetts, United States of America; Fred Hutchinson Cancer Center, United States of America

## Abstract

**Background:**

In Latin American countries over-the-counter (OTC) dispensing of antibiotics is common. In 2010, both Mexico and Brazil implemented policies to enforce existing laws of restricting consumption of antibiotics only to patients presenting a prescription. The objective of the present study is therefore to evaluate the impact of OTC restrictions (2010) on antibiotics consumption in Brazil and Mexico.

**Methods and Findings:**

Retail quarterly sales data in kilograms of oral and injectable antibiotics between January 2007 and June 2012 for Brazil and Mexico were obtained from IMS Health. The unit of analysis for antibiotics consumption was the defined daily dose per 1,000 inhabitants per day (DDD/TID) according to the WHO ATC classification system. Interrupted time series analysis was conducted using antihypertensives as reference group to account for changes occurring independently of the OTC restrictions directed at antibiotics. To reduce the effect of (a) seasonality and (b) autocorrelation, dummy variables and Prais-Winsten regression were used respectively.

Between 2007 and 2012 total antibiotic usage increased in Brazil (from 5.7 to 8.5 DDD/TID, +49.3%) and decreased in Mexico (10.5 to 7.5 DDD/TID, −29.2%). Interrupted time series analysis showed a change in level of consumption of −1.35 DDD/TID (p<0.01) for Brazil and −1.17 DDD/TID (p<0.00) for Mexico. In Brazil the penicillins, sulfonamides and macrolides consumption had a decrease in level after the intervention of 0.64 DDD/TID (p = 0.02), 0.41 (p = 0.02) and 0.47 (p = 0.01) respectively. While in Mexico it was found that only penicillins and sulfonamides had significant changes in level of −0.86 DDD/TID (p<0.00) and −0.17 DDD/TID (p = 0.07).

**Conclusions:**

Despite different overall usage patterns of antibiotics in Brazil and Mexico, the effect of the OTC restrictions on antibiotics usage was similar. In Brazil the trend of increased usage of antibiotics was tempered after the OTC restrictions; in Mexico the trend of decreased usage was boosted.

## Introduction

Inappropriate use of antibiotics enhances the development of antibacterial resistance, which is an important public health issue. It leads to treatment failures causing deaths and an increase in use of more costly antibiotics [1], [2]. In many of the Latin American countries, prohibition of over the counter (OTC) sales of antibiotics in private pharmacies is not enforced, and self-prescription with antibiotics is common [3] because antibiotics are still requested and sold without prescription in private pharmacies. During previous years, various countries implemented policies to enforce prohibition of OTC antibiotic sales. Chile was one of the first countries in the region documenting the implementation of this type of policy that took place in September 1999, which resulted in a notable decrease in the short-term consumption [4], nevertheless it slowly increased from 2002 onwards [5].

Other Latin American countries have followed Chile's example over the past years. In 2005, Colombia started to regulate the OTC sales of antibiotics only in the capital city Bogota, while in Venezuela at the beginning of 2006, a similar policy was implemented but only applied to three therapeutic groups: macrolides, quinolones and third generation cephalosporins. The effect of these policies were evaluated recently, showing a decrease in level of consumption in Colombia, but no change in level or trend in Venezuela [6]. Ultimately, two of the largest countries in Latin America, Brazil and Mexico, implemented a similar policy during 2010 enforcing the prohibition of all systemic antibiotic sales without prescription.

For many years Mexico had the highest antibiotic consumption in the region [7]. The antibiotics as therapeutic group have occupied the second place in retail sales (40% without a prescription), but the first place with regards to reports on adverse reactions [8] [9]. The consequences of self-prescription were highlighted during the epidemic of influenza A (H1N1) in 2009. Indeed, the Mexican government justified the antibiotic regulation in 2010 [10] arguing that it would prevent harmful self-medication with antibiotics that had led to delayed medical diagnosis of life-threatening complications during the influenza epidemic [9]. This regulation requires prescriptions for antibiotics to be retained and registered in pharmacies, and imposes fines to the owners of the pharmacies for non-compliance.

Brazil has been catalogued by IMS Health as a *pharmemerging* country with a pharmaceutical growth over the last few years and an increased government investment in pharmaceutical manufactures [11]. Approximately 40% of the medicines consumed in Brazil are antibiotics and they are commonly self-medicated; in 2008 alone, the sale of these medicines had a revenue of 377 million USD, with over 70 million units sold [12]. The National Health Surveillance Agency in Brazil (ANVISA) has discussed the need to improve the control of sales of antibiotics since 2009; however, it was the spread of the multi-resistant KPC bacteria (*Klebsiella Pneumoniae Carbapenemase*) and related deaths from hospital infections during 2010 that speeded up the process of carrying out the regulation [9], which was implemented in November of 2010 [13]. After that, the regulation had some modifications detailing that the pharmacies should keep a copy of the prescriptions; from April 2013, the antibiotics were included into the National Controlled Substances Management System (SNGPC) to improve the supervision of their consumption [14]. None of the two countries carried out an information campaign to prevent the inappropriate use of antibiotics, but Mexico did a campaign to inform the public about the regulatory changes.

Monitoring antibiotics consumption has been encouraged in order to design and evaluate interventions directed at optimizing the use of these medicines and prevent increasing resistance [15]. The evaluation of a policy implementation is relevant to identify its impact and take corrective actions if needed. Cross-national analysis can help to identify changes in trends of consumption in each country and understand the impact of similar measures in different settings. The aim of the present study is therefore to assess the impact of the antibiotics consumption restrictions introduced in 2010 in Mexico and Brazil and compare the effect of the measures in these two countries.

## Methods

### Data source and setting

For this study, we obtained retail quarterly sales data in kilograms of oral and injectable antibiotics in the private sector from 2007 to the first two quarters of 2012 for Brazil and Mexico by submitting a research protocol to IMS Health under their Global Health Research program explaining the objectives and methodology to conduct the present study. The database was constructed with information of manufacturers and retail wholesalers. The kilograms sold of each antibiotic was converted into a daily defined dose per 1,000 inhabitants days (DDD/TID) according to the Anatomical Therapeutic Chemical (ATC) classification system proposed by the World Health Organization [16]. Annual information on the population of Mexico and Brazil was obtained from the Pan American Health Organization records [17] and the population in each quarter was estimated using the growth rate per year.

The analysis was conducted in two stages: first, for the total amount of antibiotics consumption, and then for therapeutic sub-groups. In both countries, penicillins, tetracyclines, quinolones, macrolides and sulfonamides were the most frequently consumed therapeutic subgroups [7], and these were therefore included as separate classes. All other antibiotics were grouped as “others” for the analysis.

### Data analysis

We first conducted a descriptive analysis calculating the average consumption in the period before and after the intervention taking the consumption of the quarters corresponding to the winter season for both countries. For Brazil this corresponded to the second and third quarter of each year, while for Mexico winter season occurs during the fourth and first quarter.

Subsequently, we used interrupted time-series analysis [18] to measure the impact of the policy implementation in each country estimating changes in level and trend in antibiotics consumption after the enforcement of the regulations. For Brazil we indicated the beginning of the regulated consumption at the first quarter of 2011 since the startup of the banning of OTC of antibiotics sales was on November 29th of 2010; while for Mexico we considered the beginning of the regulated consumption at the last quarter of 2010, since the regulation took place from August 25th of the same year. For this analysis we included all data points (quarters) from the beginning of 2007 to the second quarter of 2012, except for the last quarter of 2010 for Brazil and the third quarter of 2010 for Mexico because these two periods were just partially affected by the restriction of OTC sales.

In the model, we included a reference group to account for changes in medicines consumption outside of the antibiotic regulation, such as changes in the economy and the health systems. We decided to use antihypertensive medicines since this group was not affected by the new policies and consumption does not present seasonal variation, but consumption would be affected by market growth or other external factors that we were interested in adjusting for.

All models were adjusted for seasonality by using dummy variables. This was only applied for the antibiotics group and conducted separately in the segments before and after intervention, because it was observed graphically that the seasonality patterns changed after the intervention. Autocorrelation was corrected using Prais-Winsten regression [19] and the Durbin Watson test of all models showed that no autocorrelation persisted. All analysis were executed using the STATA Software version 12 [20].

## Results

**Figure 1:**
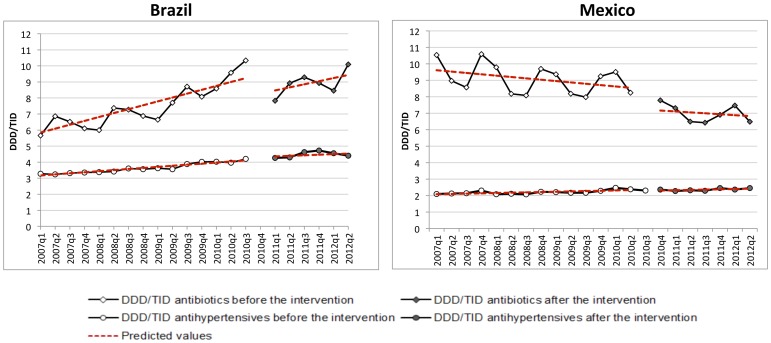
Trends in consumption in DDD/TID for Brazil and Mexico (2007–2012).

**Figure 2:**
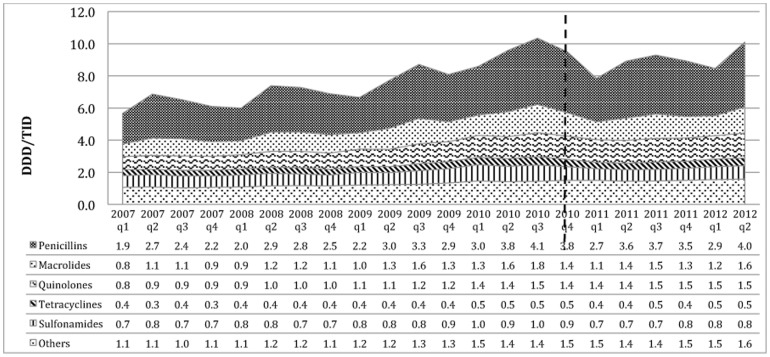
Consumption of antibiotics therapeutic subgroups in Brazil. The dotted line represents the beginning of the regulation to prohibit the OTC sales of antibiotics.

Between January 2007 and June 2012 total antibiotics usage increased in Brazil (from 5.7 to 8.5 DDD/TID, +49.3%) and decreased in Mexico (10.5 to 7.5 DDD/TID, −29.2%) in the private sector. In each year, we observed that Brazil had the highest consumption during the second and third quarter while Mexico had the highest consumption between the fourth and first quarter. For both countries this corresponds to their respective winter seasons. Antihypertensive medicines did not show seasonality in consumption as was expected. In Brazil we observed that for both therapeutic groups, antihypertensives and antibiotics, the consumption increased during the study period. The slope for antihypertensives was 0.071 DDD/TID per quarter between 2007 and 2012 while the slope was 0.18 DDD/TID per quarter for antibiotics in the same period. In Mexico, the antihypertensives showed an *increase* in consumption (slope = 0.016 DDD/TID per quarter) and the antibiotics had a negative trend of −0.15 DDD/TID per quarter (Figure 1). In addition to this, we observed a difference in seasonal patterns after intervention in both countries, the difference in consumption between summer and winter was smaller after the regulation started, particularly for Mexico (Figures 1 and 2).

Interrupted time series analysis adjusting for antihypertensive consumption showed a change in level of consumption of −1.35 DDD/TID (p<0.01) for Brazil (Table 1) and −1.17 DDD/TID (p<0.00) for Mexico (Table 2), without a significant change in the trend after the regulation was implemented (Figure 1). The change in level for Mexico was only significant after the adjustment for seasonality.

**Table 1:** Results of the time series analysis of Brazil for all antibiotics and by therapeutic sub-group.

	All	Tetracyclines	Penicillins	Sulfonamides	Macrolides	Quinolones	Others
**Intercept**	5.604 (0.000)	0.314 (0.000)	2.033 (0.000)	0.667 (0.000)	0.852 (0.000)	0.745 (0.000)	0. 973 (0.000)
**Change in level after intervention DDD/TID^¥^**	**−1.348 (0.005)**	−0.256 (0.113)	**−0.635 (0.018)**	**−0.414 (0.023)**	**−0.465 (0.010)**	−0.169 (0.328)	−0.163 (0.348)
**Change in trend after intervention DDD/TID per quarter^¥^**	−0.018 (0.798)	0.036 (0.375)	0.012 (0.814)	0.326 (0.430)	0.023 (0.568)	0.006 (0.885)	0.022 (0.596)
**Rˆ2**	0.967	0.995	0.925	0.993	0.993	0.992	0.992

¥Adjusted for reference group, p values in parentheses. All: all therapeutic groups combined.

**Table 2:** Results of the time series analysis of Mexico for all antibiotics and by therapeutic sub-group.

	All	Tetracyclines	Penicillins	Sulfonamides	Macrolides	Quinolones	Others
**Intercept**	9.695 (0.000)	1.130 (0.000)	3.538 (0.000)	1.390 (0.000)	0.966 (0.000)	1.073 (0.000)	1.582(0.000)
**Change in level after intervention DDD/TID^¥^**	**−1.172 (0.001)**	0.008 (0.958)	**−0.857 (0.000)**	−0.174 (0.068)	0.049 (0.622)	−0.041 (0.647)	0.131 (0.141)
**Change in trend after intervention DDD/TID per quarter^¥^**	0.026 (0.663)	0.015 (0.477)	0.002 (0.942)	0.022 (0.145)	−0.008 (0.662)	−0.006 (0.687)	0.005 (0.722)
**Rˆ2**	0.995	0.975	0.962	0.990	0.989	0.987	0.973

¥Adjusted for reference group, p values in parentheses. All: all therapeutic groups combined.

**Figure 3:**
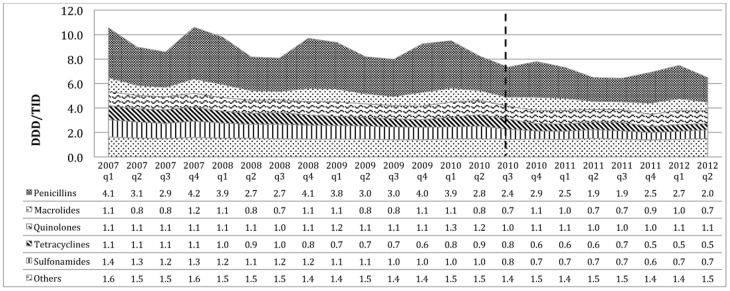
Consumption of antibiotics therapeutic subgroups in Mexico. The dotted line represents the beginning of the regulation to prohibit the OTC sales of antibiotics.

Penicillins were the most frequently consumed antibiotic in both countries (see Figures 2 and 3). Prior to the intervention, the average consumption of penicillins in Brazil was 3.1 DDD/TID (which represents 39% out of total antibiotics consumption). After the intervention in Brazil, the consumption had a significant decrease in level of −0.64 DDD/TID (p<0.00), a non-significant change in trend of 0.012 DDD/TID per quarter (p = 0.814) and an average consumption of 3.8 DDD/TID, but this decrease did not have an impact in the proportional consumption (40% out of the total) since the total consumption trend remained increasing. In Mexico this therapeutic sub-group had a consumption of 4.0 DDD/TID (41% out of total) before the intervention with a significant decrease in level after the intervention of −0.86 DID/TID (p<0.00), with no change in trend (0.002 DDD/TID per quarter, p = 0.942) and an average consumption of 2.7 DDD/TID corresponding to the 36% out of the total consumption after the policy intervention.

Quinolones had the same consumption in both countries before the regulations started, 1.1 DDD/TID (14% out of the total consumption in Brazil and 12% out of the consumption in Mexico). The mean consumption in Brazil for this group increased by 0.4 DDD/TID meaning a 2% increase relative to the total consumption and did not change in absolute values with a no significant change in level of consumption of −0.17 DDD/TID (p = 0.328) and a non-significant change in trend of 0.006 DDD/TID per quarter (p = 0.885). For Mexico, even though the absolute consumption did not change, it increased relatively by 3% out of the total consumption; neither the change in level and trend were statistically significant −0.041 DDD/TID (p = 0.647) and −0.006 DDD/TID per quarter (p = 0.687) respectively.

Macrolides had an increase in the absolute consumption of 0.2 DDD/TID in Brazil with a consumption of 1.5 DDD/TID after the regulation, representing a relative reduction of 1%, with 16% out of the total consumption. A significant change in level for this group was −0.47 DDD/TID (p = 0.010) and a non-significant change in trend of 0.02 DDD/TID per quarter (p = 0.568). The absolute consumption in Mexico of this therapeutic group had a reduction of −0.1 DDD/TID having a consumption of 1 DDD/TID after the regulation and representing a relative increase of 2% out of the total consumption with a change in level of 0.049 DDD/TID (p = 0.622) and a change in trend of −0.008 DDD/TID per quarter (p = 0.662).

The mean consumption of sulfonamides in Brazil did not change in absolute numbers having a 0.8 DDD/TID of consumption before and after the regulation, but represented a relative decrease of 2% out of the total consumption during the time studied. The change in level was statistically significant with a decrease of 0.44 DDD/TID (p = 0.023) and a no significant change in trend of 0.33 DDD/TID per quarter (p = 0.430). Mexico had a reduction of consumption of 50% in the absolute consumption of this sub-group from 1.2 to 0.6 DDD/TID, and a relative reduction of 3% out of the total consumption with a significant reduction of the level of 0.17 DDD/TID (p = 0.068) and a non-significant change in trend of 0.022 DDD/TID per quarter (p = 0.145).

## Discussion

The main objective of this study was to estimate the impact of the OTC regulation on the antibiotics consumption in Brazil and Mexico in the private sector using retail data. We showed that after the regulations took place, an immediate and similar decrease of around 1 DDD/TID in the level of antibiotics consumption was seen in the private sector in both countries, despite the different consumption patterns before the implementation of these policies. We also tested whether the policy was associated with a reduction in the consumption of the therapeutic groups; a significant change in level of consumption by therapeutic group was only observed for the penicillins and sulfonamides in both countries, and macrolides just in Brazil. Unexpectedly, we did not find statistical significant changes in trend of the total consumption or any of the therapeutic subgroups.

A previous study assessing the trend in consumption for Mexico and Brazil between 1997 and 2007 showed a decrease in consumption in Mexico and a stable consumption in Brazil [7]. Our study shows that this decrease in antibiotics consumption in Mexico continued after 2007. However, we found that Brazil is having an increase in consumption in the private sector between 2007 and 2012, and the regulation did not affect this trend. Assuming a relatively stable prevalence of bacterial infections, one could assume that the antibiotics consumption in Brazil would also be constant or decreasing for those antibiotic groups that were the mostly demanded without prescription before the regulation. Further analysis is needed to explore the factors such as the economic growth that could be contributing to the increase in antibiotics sales in this country as well as to assess the effects of increased sales on antibacterial resistance. By including antihypertensives as a control group, we ruled out the potential effects of a general change in consumption in the private sector such as the shifting from the public to the private sector or changes in consumption of pharmaceuticals due to economic changes in both countries.

In this study we calculated the consumption using retail (private sector) data for two reasons. First, the change in regulation on OTC sales could have a greater repercussion in the private sector given that in the public sector a prescription was needed to get medicines even before the regulation. Therefore, self-medication with antibiotics is less common in that sector [6]. Second, there are no other sources of information to calculate and compare the antibiotics consumption between countries because of the inexistent uniform databases between countries to conduct a similar analysis. To enable better comparisons of total drug consumption, we calculated the DDD/TID using the whole population of each country as denominator. Therefore, actual use of antibiotics in the whole country (including the public sector) is higher than the consumption found in the present work. According to IMS Health reports, pharmaceutical volume coverage was 46% for Mexico and 72% for Brazil [21].

The effect of regulating OTC sales of antibiotics in Brazil and Mexico was smaller than the impact found in Chile (−5.56 DDD/TID) but similar to the effect reported in Colombia (−1 DDD/TID) [6]. However it is important to mention that in Colombia the regulation only took place in the capital city, therefore we were expecting a bigger impact in Mexico and Brazil where the regulation took place throughout the country as in Chile. The differences observed between countries could be due to many factors, for example: the regulation implemented in Chile during 1999 was reinforced with an educational campaign and involvement of pharmacists. However these actions were not sustained and possibly because of this the consumption started to increase since 2002 [5]. However, we found no description of a nationwide campaign to promote appropriate use of antibiotics at the time that both regulations took place for Brazil and Mexico. In Mexico the government informed the public about the regulatory changes. In addition, in Mexico physician offices were installed within or right next to pharmacies only separated by a wall; pharmacies' customers demanding antibiotics OTC are referred to these physicians' offices to get a prescription. There is not yet evidence about antibiotics prescribing patterns by these offices; but, since their installment was explicitly recommended by market consultancy groups in Mexico as a way “to avoid losses from antibiotics sales” [9], it is probable that prescriptions for antibiotics issued in these offices partly compensated for OTC sales of antibiotics. Although in Brazil a copy of the prescription for an antibiotic is required to be retained; however, there are anecdotal reports of problems in the verification of the prescription retention, and thus the policy might not be fully implemented. Therefore, from April 2013 onwards antibiotics are included into the National Controlled Substances Management (SNGPC) to improve the monitoring of their consumption. Pharmacies nationwide must submit information electronically concerning drugs subject to the reporting national system [14]. The SNGPC is an important regulatory tool for monitoring drug use nationally and played a key role in the removal of some appetite suppressant drugs from the Brazilian market when the data confirmed abuse [22]; thus, further changes in antibiotics consumption might be observed from 2013.

In the present study, in both countries, the therapeutic group of penicillins was the group with the major consumption and had the highest contribution to the seasonality patterns observed in the total consumption. High seasonal fluctuations in antibiotics consumption suggest inadequate use for viral acute respiratory tract infections (ARI) [23]. Graphically, we observed a change in the seasonality patterns after the policy started, particularly for Mexico, with less difference between consumption in winter and summer than before the regulations started. Penicillins has been reported to be the most common group for self-medication in Mexico [7]. The observed effect on seasonality may be due to the reduction of self-medication with antibiotics for acute respiratory infections. This seems to be confirmed by the fact that we found only a very small change in the overall percentage of consumption of each of the therapeutic groups. The reduction in the percentage of penicillins (5%) out of total antibiotic consumption was replaced by macrolides and quinolones.

More work is required to generate evidence on how to develop an appropriate and effective policy to reduce inappropriate antibiotics consumption in the context of health system reforms in Latin America, where barriers to access to medicines for the poor population, economic crisis, and inadequate prescription and self-medication practices place important challenges. Even though regulating sales of antibiotics is relevant to promote appropriate use, it is only one component of a more comprehensive strategy that is required; campaigns targeting public promoting appropriate use of antibiotics and interventions directed to medical staff are also important to ensure adequate antibiotics consumption. Implementing monitoring systems to track the implementation of the regulation in terms of consumption, antibiotic resistance and infections rates are also core components of a more comprehensive strategy.

## Conclusions

Despite different overall usage patterns of antibiotics in Brazil and Mexico, the effect of the policy enforcing OTC restrictions on antibiotics usage was similar. In Brazil the trend of increase usage of antibiotics was tempered after the OTC restrictions, in Mexico the trend of decreased usage was boosted. The reinforcement of regulations banning the OTC sales of antibiotics need to be monitored together with the development of more comprehensive measures to promote adequate utilization of antibiotics in both countries.
